# The Successful Management of an Adult Patient With Unrepaired Tetralogy of Fallot for Caesarian Section: A Case Report From South Africa

**DOI:** 10.7759/cureus.49689

**Published:** 2023-11-30

**Authors:** Kirsten Morley-Jepson, Mithasha Gayaparsad, Oliver Smith

**Affiliations:** 1 Anaesthesiology, University of the Witwatersrand, Johannesburg, Johannesburg, ZAF

**Keywords:** tetralogy of fallot, cardiac pathology in obstetrics, obstetric regional anaesthesia, cardiac for non-cardiac surgery, cardiac ct congenital heart diseases, loco-regional anaesthesia

## Abstract

Tetralogy of Fallot (TOF) is a congenital heart defect characterized by four interlinked cardiac anomalies, leading to cyanosis and significant health risks if left untreated. We present the management and outcome of an adult patient with unrepaired TOF at 34 weeks of pregnancy at a central Johannesburg hospital. Uncorrected TOF in adulthood, especially in the late stages of pregnancy, is rare, presenting challenges for both the mother and baby. This case report outlines our successful management strategy, highlighting the importance of careful hemodynamic stability.

The primary goal in managing this complex case was to prevent significant changes in systemic vascular resistance (SVR) and pulmonary vascular resistance (PVR). We diligently avoided triggers for worsening right-sided pressures and aimed to maintain haemodynamic stability and therefore optimise oxygen delivery and minimise blood loss and dehydration. Our approach focused on preventing a worsening right-to-left shunt, which exacerbates hypoxia and haemodynamic instability.

Regarding the anaesthetic technique, we discuss the advantages and disadvantages of both general and regional anaesthesia. While there is no gold standard, the choice should be individualized based on the patient's condition and treating unit practice. We describe the use of a graded epidural anaesthesia technique, which proved to be a safe and effective method for managing a parturient with significant cardiac disease during caesarean section. This technique resulted in minimal hemodynamic changes and superior post-operative pain control and avoided potential side effects associated with general anaesthesia. Notably, the technique relied solely on a neuraxial anaesthetic technique, minimising the risk of neonatal cardiopulmonary depression.

This case report serves as the first documented instance from South Africa of successfully anaesthetizing an adult patient with unrepaired TOF in late pregnancy for a caesarean section. The graded epidural technique emerged as a secure option for anaesthetic management in a challenging case, providing important insights into the care of patients with complex cardiac conditions during pregnancy.

## Introduction

Tetralogy of Fallot (TOF) is the commonest known cyanotic congenital heart defect [1]. First diagnosed by French physician Arthur Fallot in 1888, it is a condition thought to be caused by incomplete rotation and abnormal division of the conotruncus of the embryonic heart [2]. Usually diagnosed in early life, it is characterized by four interlinked cardiac defects, namely pulmonary stenosis (PS), ventricular septal defect (VSD), overriding aorta, and right ventricular hypertrophy (RVH) [3]. Corrective surgery should occur in infancy to reduce the risk of sequelae including thromboses, endocarditis, and cardiac failure [4].

Allowance of this congenital cardiac defect to progress to adulthood severely worsens patient physiology and complicates the repair [4]. Addition of the physiological ramifications of pregnancy especially in the latter stages presents a markedly precarious maternal clinical condition which requires meticulous obstetric and anaesthetic care [5]. 

We present our management of an adult patient with unrepaired TOF at 34 weeks of pregnancy, at a central Johannesburg hospital.

## Case presentation

A 24-year-old female, G2P0M1, presented to the obstetrics department outpatient clinic at a tertiary hospital in Central Gauteng with an unrepaired TOF at 31 weeks of gestation. The reason for her presentation was premature, prelabour rupture of membranes (PPROM). She initially reported an otherwise uneventful pregnancy. However, on further enquiry, the patient had noted a general decline in effort tolerance in keeping with the progression of her pregnancy, with occasional episodes of increasingly severe shortness of breath and palpitations, which resolved with rest. She did not give a history of characteristic cyanotic spells associated with TOF. She was not on medication for her primary cardiac lesion.

The patient was diagnosed with TOF in her first year of life. She was never scheduled for repair due to defaulting hospital care by her mother. According to the patient, she was not made aware of this diagnosis until the age of 12 when she presented to the hospital with symptoms of shortness of breath on exertion. She again was lost to follow up only to represent during her first pregnancy. This occurred in 2020 at age 22. The pregnancy was terminated at 12 weeks of gestation for medical reasons relating to her congenital heart defect. She required no surgical or anaesthetic management. She was then scheduled for corrective surgery; however, the patient was once again lost to follow-up. Additional medical history includes a diagnosis of human immunodeficiency virus (HIV) for which she is on highly active antiretroviral therapy. Apart from severe wasting, which is likely multifactorial in aetiology, the patient had no clinical stigmata of advanced HIV.

A general examination of the patient revealed marked central cyanosis with digital clubbing, a body mass index (BMI) of less than 20 kg/m^2^ and a Glasgow coma score (GCS) of 15/15. She had a pan-systolic murmur heard throughout the precordium, with an associated thrill. She had no features of respiratory distress at rest and no other features of acute congestive cardiac failure. The patient was saturating at 78-80% on room air with a partial pressure of oxygen on a room air arterial blood gas (PaO2) of 47.8 mmHg.

**Figure 1:**
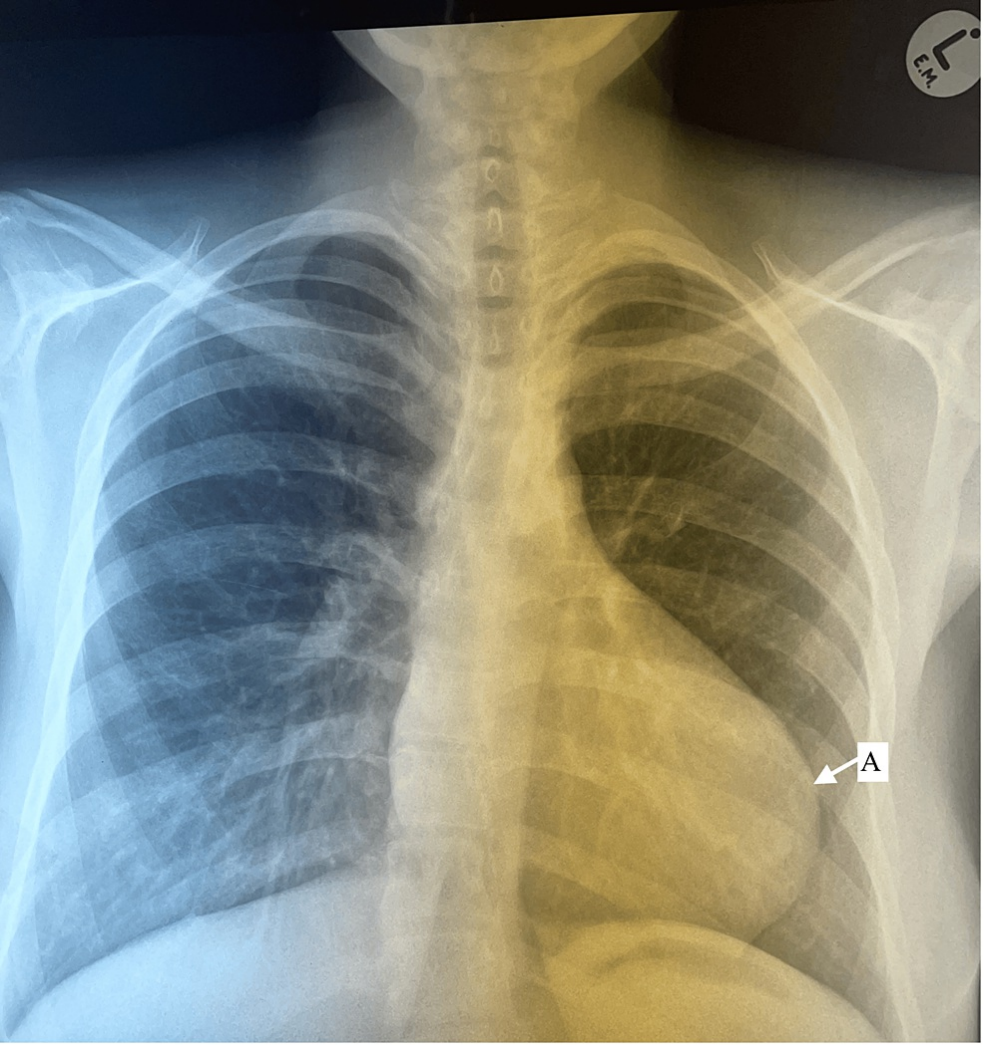
PA Chest X-ray demonstrating the characteristic ‘boot-shaped’ heart (A)

The patient’s electrocardiogram revealed right axis deviation with right ventricular hypertrophy (RVH). Her chest X-ray showed a boot-shaped, hypertrophic cardiac shadow with poor lung vascularity (Figure 1).

**Figure 2:**
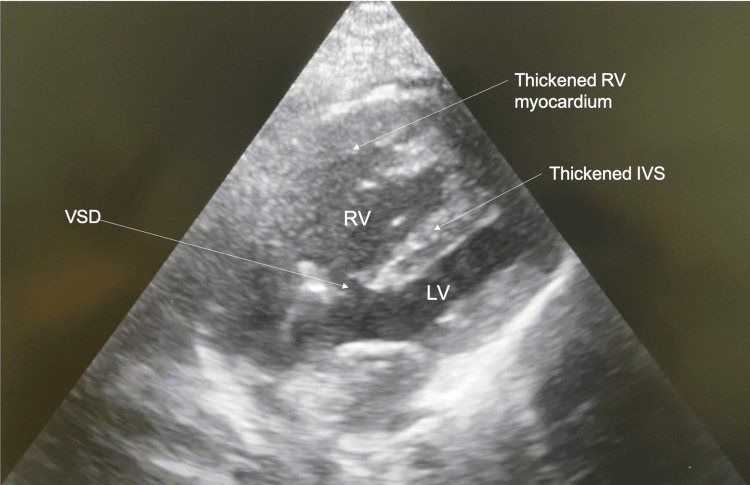
Trans-thoracic echo - apical four-chamber view LV: Left ventricle; RV: right ventricle; IVS: intraventricular septum; VSD: ventricular septal defect

**Figure 3:**
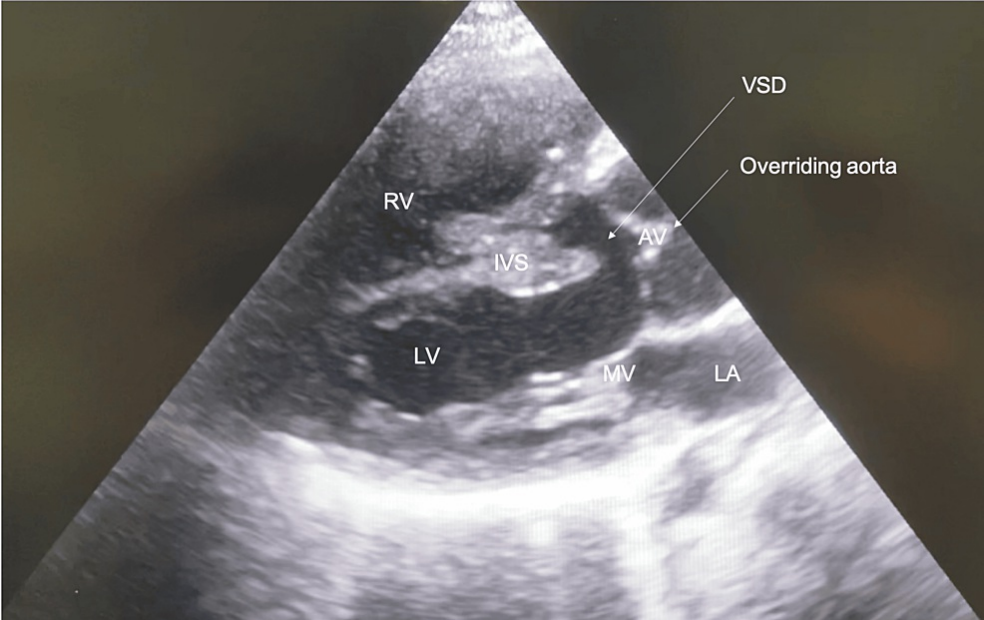
Trans-thoracic echo - parasternal long-axis view LA: Left atrium; MV: mitral valve; LV: left ventricle; RV: right ventricle; IVS: intraventricular septum; AV: aortic valve; VSD: ventricular septal defect

**Figure 4:**
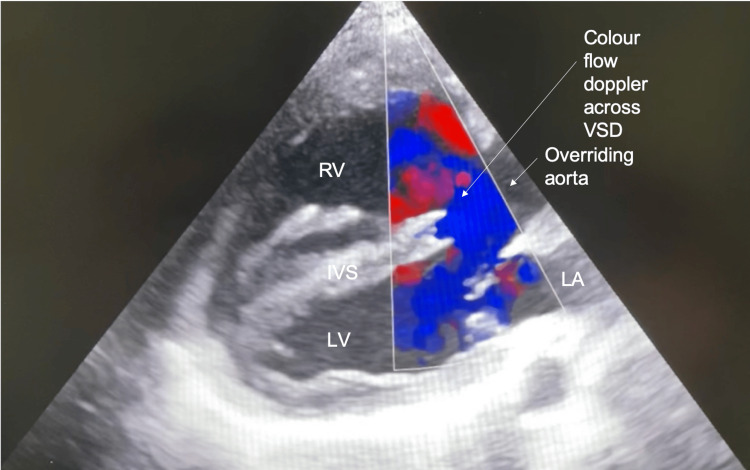
Parasternal long-axis view with colour flow Doppler across a perimembranous ventricular septal defect LA: Left atrium; LV: left ventricle; RV: right ventricle; IVS: intraventricular septum; VSD: ventricular septal defect

**Figure 5:**
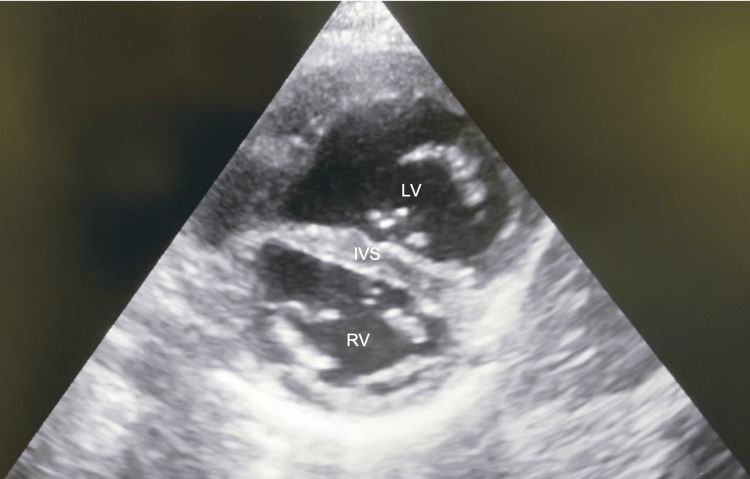
Trans-thoracic echo - parasternal short-axis view highlighting a straight intraventricular septum LV: Left ventricle; RV: right ventricle; IVS: intraventricular septum

**Figure 6:**
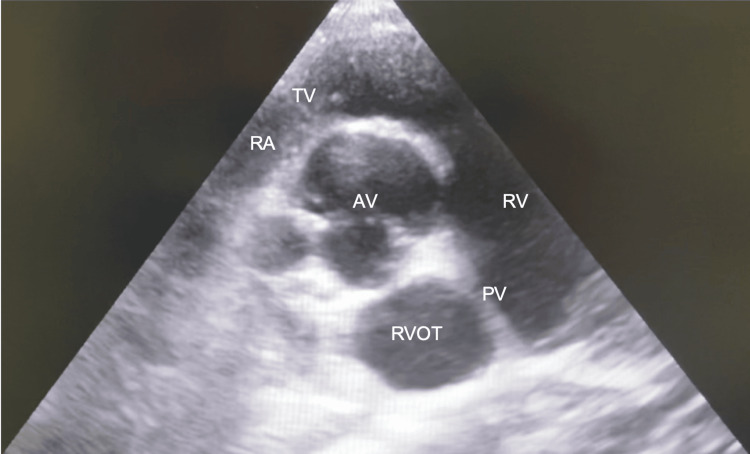
Trans-thoracic echo - parasternal aorta short-axis view highlighting a narrowed pulmonary valve AV: Aortic valve; RA: right atrium; TV: tricuspid valve; RV: right ventricle; PV: pulmonary valve; RVOT: right ventricular outflow tract

On echocardiography, the patient displayed all the features of a congenital TOF: pulmonary stenosis of the pulmonary valve and infundibulum with significant RVH (free wall measured 14.6mm); aortic override of 50%, with the patient’s aorta seen to override both right and left ventricles equally and a large peri-membranous VSD with bidirectional shunt was seen. No ASD was seen. The right ventricular outflow tract (RVOT) pressure gradient measured 30mmHg. The patient’s pulmonary valve annulus was measured to be approximately 12 mm. The patient’s RV systolic function was good with a tricuspid annular plane systolic excursion measurement of 20.5mm. Tricuspid regurgitation was not mentioned. There was good left ventricular systolic and diastolic function with an ejection fraction of 76.1%. The E/A ratio was 0.98 and E/e ratio was 3.92 (Figures 2-6).

At this point, she was treated for PPROM with antibiotic therapy, tocolytic agents, and two doses of steroid therapy for foetal lung maturation. 

A multidisciplinary team meeting comprising members of the obstetric, intensive care, paediatric and anaesthetic departments, was held at this time to discuss the optimum timing of delivery prioritising safety for mother and foetal viability. Thus at 31 weeks, it was decided to allow her pregnancy to progress to 34 weeks of gestation, considering the patient was not in cardiac failure and in order to maximise foetal maturity. Taking into account options for delivery, an elective caesarean section was planned. 

The patient was extensively counselled regarding all details of the anaesthetic plan including peripheral intravenous access, awake insertion of invasive lines, neuraxial insertion as well as possible complications, and changes to the plan that may occur intra-operatively. Informed consent was given by the patient.

Considering the pathophysiology of the patient’s unrepaired cardiac lesion, a slowly graded epidural anaesthetic technique was planned. At 34 weeks of gestation, the patient arrived at the theatre for her caesarean delivery. Her baseline vitals were as follows: a HR of 81 bpm, BP of 118/70 mmHg and her room air saturation was 78%. The presence of a foetal heartbeat was confirmed with Doppler.

Two intravenous peripheral lines were inserted (14G and 20G) and the patient was preloaded with 500ml of hydroxyethyl starch (HES), specifically Volulyte colloid solution. Surgical antibiotic prophylaxis and prophylaxis for infective endocarditis were achieved with the administration of 1.2g amoxicillin-clavulanic acid. Her baseline saturation improved modestly to 80-82% on nasal prong oxygen, flowing at a rate of four litres per minute. 

Whilst observing sterile techniques, an ultrasound-guided right internal jugular central venous catheter and a landmark-guided left radial arterial line were inserted and transduced for arterial and central venous pressure (CVP) monitoring. An epidural catheter was then inserted with the patient in the sitting position, at a spinal level of L3/L4, also under sterile conditions.

In order to achieve our haemodynamic goals, a 200:1 phenylephrine infusion was initiated prior to dosing the epidural at a low dose of 0.02ug/kg/min and titrated to the patient’s haemodynamic targets which were to maintain MAP within 10% of her starting baseline MAP. The maximum dosage used was 0.2ug/kg/min. This was carefully monitored using arterial line tracing. The CVP was also monitored and the target was also to keep this value within 10% of starting CVP. Phenylephrine was used to combat the vasodilatory effects of the epidural and was the preferred agent for this case as the patient did not require ionotropy. Secondly, should the patient have suffered infundibular spasm, phenylephrine would assist in treating this complication of TOF. 

A test dose of 2ml 2% lignocaine with adrenaline 1:200 000 was given to rule out intrathecal or intravascular catheter placement. However, consideration should be given to this action, as in a high-risk patient, if placement had been intrathecal it would result in a precipitous drop in SVR. Thus retrospectively, authors encourage other methods to rule out incorrect placement such as using low dose and volume of bupivicaine or lower concentration of lignocaine followed by observation to confirm placement. 

Epidural loading began with incremental injections of 10-15mg plain (2-3ml 0,5%) bupivacaine at five-minute intervals. The level of the epidural block was confirmed with an ice pack prior to the next bupivacaine dose. A total of 60mg 0.5% bupivacaine (12ml) injected incrementally over 30 minutes achieved a suitable level for the caesarean section (S4-T4).

Surgery commenced and a live male infant with normal Apgar scores was delivered. An intravenous oxytocin bolus of 1IU was given over 2 minutes to avoid a drop in SVR and a low-dose infusion of 20 IU in 200ml normal saline was started. It was run at 2.5 IU per hour. Low-dose bolus and infusion were used to avoid a drop in SVR and increasing PVR. Twenty minutes into the procedure, top-up dosing was required signalled by recession of the epidural level. Thus, a further 20mg bupivacaine was administered via the epidural catheter in divided doses of 10mg (2ml) each at 15-minute intervals to facilitate the completion of the surgery.

Following completion, a 1mg/ml (0,1%) bupivacaine epidural infusion was started at 4ml/hr and 1.5mg morphine was administered into the epidural space to provide prolonged post-operative analgesia. The phenylephrine infusion was gradually weaned as the dense epidural level regressed. There were no episodes of respiratory distress or cyanotic spells in the perioperative period. 

In addition to careful blood pressure monitoring using the invasive blood pressure monitor, CVP was also monitored and maintained as close to baseline as possible (7-9mmHg). This was done with targeted fluid administration as well as avoidance of any precipitants of increasing pulmonary resistance such as hypoxia, hypercarbia, acidosis and sympathetic stimulation. 

At the completion of the surgery, total blood loss was recorded as 550ml. The total fluids received were 500ml volume and 650ml Ringers lactate. The urine output recorded was 360ml. 

The patient was transferred to the intensive care unit (ICU) with the following vitals: HR 78 bpm; BP 115/76 mmHg and saturations of 82% on nasal cannula. She remained GCS 15/15 throughout. The epidural 0.1% bupivacaine infusion was continued to provide analgesia in the ICU for a further 24 hours. Thereafter the epidural was removed and her analgesia was managed by the ICU team through a combination of oral and intravenous agents. The patient was discharged from ICU on day 2 post-operatively and home on day 4 post-operatively. 

## Discussion

TOF is the commonest cyanotic congenital heart defect diagnosed in infants [1]. As described by Fallot in 1888, TOF is characterised by four interlinked cardiac defects which may vary in severity [2]. These comprise a VSD, obstructed RVOT, overriding aorta, and RVH [2]. These changes from normal cardiac anatomy result in deoxygenated blood mixing into the systemic circulation. Output from the RV is directed through the VSD and then through the overriding aorta, instead of normal flow through the RVOT toward the lungs for oxygenation [3]. The gradient created from the high-pressure hypertrophic and obstructed RV to the unusually lower pressure LV augments this pathology [3]. Systemic hypoxia, polycythaemia, coagulopathy, and altered acid-base status as well as episodic and reactive pulmonary vasoconstriction can result [5]. The severity of these sequelae depends on the size of the VSD, degree of pulmonary stenosis and integrity of the right ventricle [5].

The amount of blood shunted and the consequent severity of the clinical picture is a dynamic process affected by the interplay of the patient’s PVR with SVR [5]. The pressures in these systems can vary significantly depending on various physiological parameters such as blood oxygen and carbon dioxide concentrations, blood pH, circulating catecholamines and temperature as well as, pharmacological parameters such as the action of vasoactive drugs [5].

If uncorrected, 70% of people born with TOF do not survive past ten years of age. Thus, survival to child-bearing age with an uncorrected lesion and presentation in an advanced stage of pregnancy is rare [6]. Pregnant patients with uncorrected TOF present significant risks for both mother and foetus including miscarriage, foetal growth restriction, foetal death and maternal death [7]. 

The reason for this increased mortality is related to the interplay of the TOF anatomy with the physiological changes of pregnancy. Normal hormonal changes in pregnancy reduce SVR and together with an increase in blood volume, also normal in pregnancy, lead to an increased cardiac output. In a patient with TOF, this can augment the right to left shunt, worsening the degree of hypoxia, polycythaemia and cyanosis and therefore maternal and foetal condition [2,3]. Therefore when the pathological effects of the TOF anatomy are combined with those of normal pregnancy, the effects are potentially catastrophic for the affected parturient. As defined by the World Health Organization (WHO) Classification of Maternal Cardiovascular Risk, these patients fall into Class III. As per this classification, these patients are at significantly increased risk of maternal mortality or severe morbidity. Expert counselling is required and if pregnancy is decided upon, intensive specialist cardiac and obstetric monitoring is needed throughout pregnancy, childbirth and the puerperium [8].

Thus, our haemodynamic goals focused on the prevention of a fall in SVR and an excessive rise in PVR. To achieve this, we avoided triggers of infundibular spasm (hypoxaemia, hypothermia, acidosis, sympathetic stimulation, elevated carbon dioxide); maintained a stable heart rate; optimised oxygen delivery and attempted to minimise blood loss and dehydration. Furthermore, a decline in SVR was managed conscientiously to avoid worsening the existing right-to-left shunt [9]. This was done by careful titration of vasopressor infusion, in our case low dose phenylephrine. This agent was chosen due to the known sequelae of neuraxial anaesthesia, specifically sympathectomy, resulting in vasodilation. our patient had a good ejection fraction and did not require inotropic support and thus phenylephrine was the agent of choice. Another pertinent step in avoiding a drop in SVR was the careful administration of oxytocin, a drug known to cause peripheral vasodilation and in addition to this it may even result in increased PVR [10]. 

In terms of anaesthetic techniques, both general and regional anaesthetic approaches have been employed in other case reports. There is no gold standard, and a risk-benefit analysis should be used and individualised to the patient’s presenting condition. Traditionally, general anaesthesia may be preferred in patients who would not tolerate a sudden sympathectomy following neuraxial anaesthesia. Such vulnerable populations include those with severe cardiac pathology, poorly performing RVs, severe pulmonary hypertension and unrepaired cyanotic congenital heart lesions, such as our patient.

However, a general anaesthetic is not without disadvantages. Some of which include a potential decrease in SVR due to vasoplegic effects of anaesthetic drugs, and a rise in PVR due to periodic hypoxia, hypercarbia, acidosis, and hypothermia [7]. Furthermore, the effects of positive pressure ventilation on the pulmonary vasculature. Specifically, this can cause reduced venous return to the right atrium due to raised intra-thoracic pressure, subsequent reduced right ventricular output and reduced pulmonary blood flow which could be disastrous in an already strained right heart as in this cyanotic lesion [7]. Additionally, the transfer of drugs across the placental barrier may cause neonatal cardiorespiratory depression after delivery [6].

As illustrated by this case report, the use of a graded epidural anaesthetic technique has been shown to be a safe and effective means of managing a parturient with significant cardiac disease for caesarean section [10]. If employed judiciously there should be minimal haemodynamic changes perioperatively with the provision of superior post-operative pain control [6]. Furthermore, this technique relies wholly on local anaesthetic agents which do not cause neonatal cardiorespiratory depression [6]. It is superior to spinal anaesthesia because the slow rate at which the epidural level is achieved together with a concomitant phenylephrine infusion avoids an acute drop in SVR. Additionally, it also maintains the benefits of sustained spontaneous breathing and avoidance of the obstetric airway and potential aspiration [7]. Other complications of general anaesthesia such as reduced mother-infant bonding and increased risk of deep vein thrombosis are also avoided [11].

## Conclusions

This is the first documented case report from South Africa of the anaesthetic management of an adult patient with an unrepaired TOF, presenting in the third trimester of pregnancy for a caesarean section. A carefully graded epidural anaesthetic technique was employed achieving our haemodynamic goals. This technique also avoided the unwanted potential side effects of general anaesthesia. Thus, neuraxial anaesthesia with a graded epidural is presented as a safe option in this case for the anaesthetic management of these challenging patients. 
